# COVID-19, geopolitics and risk management: Towards framing a reciprocal, coordinated, responsive and empathetic international education sector

**DOI:** 10.1177/14782103231163480

**Published:** 2023-03-27

**Authors:** Ly Thi Tran, Diep Thi Bich Nguyen, Jill Blackmore, Baogang He, Huy Quan Vu

**Affiliations:** School of Education, 2104 Deakin University, Australia; School of Humanities and Social Sciences, 2104 Deakin University, Australia; Department of Information Systems and Business Analytics, 2104 Deakin University, Burwood, Australia

**Keywords:** Geopolitical crisis, pandemic, international education, higher education response, student mobility, transnational education

## Abstract

Geopolitics is shaping the international education landscape. International education has trationally been used as a tool to boost transnational cooperation, foster multilateral and global ties, and reduce tensions between nations. Such a role has been eroded and international education has been weaponised in the context of escalating political turbulences and disputes over the COVID-19 pandemic. In particular, the relationship between Australia and China, with international student flows interrupted due to COVID-19, is overshadowed by escalating geopolitical tensions in the Indo-Pacific region. Based on a qualitative study, this article examines stakeholders’ views on the responses of the Australian international education sector and universities to emerging geopolitical tensions. The conjuncture of geopolitics, COVID-19 and Australia's former government responses magnified a sense of crisis for universities and the international education sector as it was at risk because of their financial reliance on international students. Based on the findings, recommendations are made for the framing of a reciprocal, coordinated, responsive and empathetic international education sector to mitigate geopolitical risks and ensure more sustainable and ethical development for the sector.

## Introduction

International education (IE) is situated in a broader political context as it involves multiple educational, social, cultural and diplomatic relationships across different countries and various fields. The key dimensions of international education, including student mobilities, international student recruitment, internationalisation of the curriculum, international research collaborations, transnational education and offshore programs, are increasingly subject to nation states’ political agendas and geopolitical turbulences. The role of international education, previously as a tool to nurture mutual understandings, intercultural learning, empathy, country-to-country harmony and peaceful cooperation, has declined (De Wit, 2020). Yang (2022) suggests that international student mobility and university’s reliance on it may be in a ‘crises of various natures – pertaining to geopolitics, economy and social reproduction of human capital’ (p. 1).

The geopoliticisation of international education over the past decade has been escalated by political movements driven by populism, national protectionism or inward-looking nationalism, anti-immigration, anti-globalisation and anti-multiculturalism (Altbach and De Wit, 2017; Hsieh, 2020). Brexit, Trump isolationism and the rise of far-right parties in Europe have fuelled the geopolitics-international education tensions and affected higher education institutions’ internationalisation agendas. Domestic politics and geopolitics cannot be disentangled.

International education has become ‘weaponised’ through these political tensions, which have been exacerbated by the COVID-19 pandemic (Ross, 2020a; Tran, 2020). COVID-19 has disrupted the international education sector and the international relationships intertwined within it. In particular, relations between Australia and China became increasingly strained as a result of escalating tensions stemming from multiple developments since 2020. The Australian government called for an international inquiry into the origins of COVID-19 and expressed its oppositional policy stance towards China in relation to issues about Hong Kong’s security law, Taiwan’s independence and China’s escalation in the South China Sea. In July 2020 the Australian government introduced new visa options for the international student cohort from Hong Kong, allowing them to extend their student visas by five years and opening new pathways to permanent residency if they met eligibility requirements (Department of Home Affairs, 2020). In response, China has deterred their students from studying or resuming to study in Australia, claiming that Australia is an unsafe and discriminatory destination (Kuo and Murphy, 2020). China has also made public a list of fourteen complaints against Australia regarding Chinese foreign investment, their position on Hong Kong, Taiwan and South China sea and media reporting which were related to Australian sovereignty and democracy (Hurst, 2021). China’s response is seen as ‘an implicit warning to other major destination countries if they do not cooperate with China’ on these above matters of concern (Tran, 2020: p. 1). In the context of the highly politicised trade boycott by China on Australian exports, international education is listed in China’s catalogue of sanctions and tariffs on Australian trade which includes beef, barley, cotton, lamb, mineral resources, seafood, sugar, timber and wine. These tensions have heightened with China not condemning the invasion of Ukraine by Russia, the AUKUS agreement between USA, UK, Australia and New Zealand, and recent moves by China seeking to develop security contracts with nations independently and collectively in the blue Pacific region (Kaboutoulaka, 2022). Yang (2022: p. 21) argues that

This awareness of the politics of crisis helps to “de-naturalise” events and developments conventionally referred to as “crises,” bringing to the fore the underlying agendas possibly pursued by different socio-political actors who respond to and leverage the said “crises.”

In the midst of such escalated tensions, Lehmann (2020) argues international education in Australia is taking a reactive approach and lacks a long-term, coordinated vision. She calls for the need to build a strategic, long-term public diplomacy strategy for Australian international education, including the focus on what she calls ‘domestic diplomacy’. That is, Australian universities and public need to be onboard to mitigate the risks for international students. However, there has been a lack of empirical evidence about how Australian universities have coordinated with related stakeholders and adapted their policies and practices to deal with the rising geopolitical crisis generally and risk management in particular (Smith and Guthrie, 2021). Furthermore, while being widely discussed in the media (e.g. The Guardian, Financial Review, ABC, the Australian and the Conversation), little research has examined geopolitics, COVID-19 and international education by taking into account the perspectives of different stakeholders.

This article responds to this critical need by providing insights into stakeholders’ perspectives on the responses from Australian government and universities to the geopolitics-COVID-19 crisis. It draws on a study that includes in-depth interviews with 22 key stakeholders including university leaders of internationalisation, and representatives from government, professional organisations and private consultancy organisations in international education. Within this article, geopolitics is understood as ‘the influence of such factors as geography, economics and demography on the politics and especially the foreign policy of a state’ (Merriam-Webster Dictionary, 2022).

## The geopolitics of international education

The relationship between international education and geopolitics is often discussed in relation to how nations draw on international education to foster mutual understandings and at the same time, exert soft power influence (Altbach and de Wit, 2017; Tran et al., 2022; Tran and Bui, 2021; Tran and Vu, 2018). Driven by the ‘education as aid’ principle, Australia sought to both support national building of developing countries in Asia and increase its positioning in the region through the introduction of the Colombo Plan in the 1950s, providing scholarships for scholars in the region to study in Australia (Tran et al., 2021a, 2021b; Tran and Nguyen, 2023). International education has been used as mechanism to advance international cooperation in higher education and build mutual understandings between nations by international education bodies such as the Institute of International Education in the US, the Deutscher Akademischer Austauschdienst in Germany and the British Council in the UK (De Wit et al., 2015). Cross-cultural or student exchange programs contribute to promoting the sense of internationalism and facilitating political and social recovery, thereby being ‘instrumental in overcoming geopolitical tensions’ (Brooks, 2015: p. 67). In delivering international education activities, education institutions such as schools and universities can therefore be considered as geopolitical sites and have a role to play in (re)shaping the geopolitical landscape. Over the past two decades, outbound mobility programs such as the ERASMUS and ERASMUS^+^ by the European Union, USA's Fulbright program and Australia’s New Colombo Plan are signature initiatives promoting public diplomacy through learning abroad (De Wit et al., 2015; Tran and Vu, 2018). The New Colombo Plan in particular embraces the Australian government’s aspiration to strengthen Australian position and engagement in the Indo-Pacific through outbound student mobility (Tran and Vu, 2018). These education exchange and learning abroad schemes underscore how outbound mobility has evolved beyond an educational experience to include public diplomacy functions shared by institution-to-institution and country-to-country connections, multilateral building and country positioning.

The development of international education and education hubs by societies like China, Malaysia, Singapore and Hong Kong, is underpinned by nation states’ desire to strengthen their leadership position in international education so as to exert their geopolitical influence (Lee, 2015). China is among the core of countries, together with the Anglophone nations states, that has actively capitalised on international education to strengthen its soft power and exert its influence regionally and globally. Central to Beijing’s use of international education as a mechanism for soft power enhancement is its ‘bring-in’ (*qing jin lai zhan lue*) strategy and return diaspora policy that aim to boost the country’s attraction as a destination for international students and establish China as a regional education hub (Cheng, 2009) and to regain the skills and talent of Chinese graduates from Western universities (Tran et al., 2021c). In particular, international education has been used strategically to support China’s political and economic agendas under the Belt and Road Initiative (BRI) (He, 2019; Tran et al., 2022). An Education Action Plan has been developed as part of the BRI that provides Silk Road Scholarship programmes, increases the quotas of inbound international students to China, and facilitates investment to boost China’s research capacity (Peters, 2020).

At the same time, China has controlled and monitored so-called Washington’s ‘peaceful evolution’ plan (He, 2013), especially in the higher education sector. It rejects American liberal arts teaching in China, discourages the use of Western English textbooks and reduces the weightage of English publications in academic promotion (Sharma, 2020; Yang, 2000). Chinese scholars must get approval before meeting their foreign counterparts, and officials must approve their papers before they present at the international conference. Higher education has fallen into a critical area for China’s comprehensive national security strategy of containing the influence of Western social sciences and humanities in China (He, 2013). This kind of securitisation has also played its role in discouraging Chinese students from studying social sciences and humanities in Western societies.

### Sino-Australian international education relationship

Prior to COVID-19, Australia’s engagement with China at all education sectors and government levels has been generally ‘positive and fruitful’ (CIE Working Group, 2019: p. 24). In 2017, Australia was the second most favourite destination for Chinese international students, after the USA (CIE Working Group, 2019). Chinese students is the largest cohort, accounting for around 30% of all international students in Australia in 2017 (DESE, 2018) and nearly 40% in 2019-2020 (DESE, 2021), with the return rate being above 78% since 2014 (China Daily, 2019). These returnee students are the important players in advancing Australia’s soft power by promoting Australian international education, maintaining academic linkages as well as professional collaboration. In terms of Australian student outbound mobility, in 2019, China was the most popular destination for Australian students. In relation to research collaboration, China has been Australia’s key partner in many important areas including advanced technological development, innovation and commercialisation at individual, institutional and governmental levels. During the 2009–2019 period, research collaboration between the two counties has soared from ‘minimal engagement’ to the point that China was Australia’s second most important research partner after the US (CIE Working Group, 2019). Between 2016 and 2018, Australia was China’s third largest partner in research collaboration based on the number of co-authored publications (ibid.).

More importantly, escalated bilateral tensions in the international relations between Australia and China has been a major blocker for Australia–China education ties. The international education relationship between Australia and China is among the most damaged one. The issues of Taiwanese independence and China’s unlawful and escalatory activities in the South China Sea (Mastro, 2021; Tyler, 2021) have proven to be thorny topics in the bilateral relationship in recent years. Throughout 2019 and 2020, the former Australian Foreign Minister, Marise Payne, as well as other representatives of the Australian government expressed concern over the situation in Hong Kong, in particular the crackdowns on citizens protesting proposed national security and extradition legislation. COVID-19 has exacerbated the geopolitical crisis between Australia and China when the Australian government called for an international independent inquiry into the origins of COVID-19 in March 2020. In 2021, both Australia and China lodged complaints with the World Trade Organisation against each other in relation to trade disputes (Haydar and Dziedzic, 2021; Starting and Clayton, 2021). The Australian government and universities have tightened security measures and overseeing international research and teaching collaborations, with significant overreach under the Morrison government, such as required reporting of changes of post-graduate thesis topics to the Minister (Ross, 2022). In the contest for South-East Asia, international education and research collaboration are key sites of Sino-Australian tensions (Gyngell, 2022).

This crisis is exacerbated by not only the pandemic but also the over-reliance of Australian universities on international students in general and Chinese students in particular for revenues to substantially subsidise its operational activities and research (Blackmore, 2020; Tran, 2020). This was further emphasised following the September 2021 announcement of the AUKUS technology-sharing defence arrangement between the United States, the United Kingdom and Australia (and the foreshadowing of Australia acquiring a nuclear propulsion submarine capability). Former Australian Prime Minister Kevin Rudd warned that ‘geopolitics could make the inflow of large numbers of Chinese students [to Australia] a collateral casualty’, and that radical market diversification was a critically needed as an insurance strategy (Prakaash, 2021).

## Research design

This article is derived from a study that explores the impacts of geopolitical turbulences on Australian international education and student mobilities. It explores stakeholders’ perspectives on how the Australian international education sector and universities have responded to geopolitical crisis. A qualitative approach allowed for an in-depth inquiry into how the complex, multi-dimensional and evolving geopolitical crisis impacts on international education practices and policy (Denzin and Lincoln, 2000). Semi-structured interviews were conducted with 22 key stakeholders involved in international education who have the expertise to comment on the impacts of geopolitics on international education. The interviewees included representatives of key organisations involved in international education in Australia, ranging from the government (2), non-government professional organisations (10), universities (6) and private consultancies (4). The participants’ demographics are summarised in Table 1. Ethics approval was sought from the university where the researchers are based. Contact details of potential participants were obtained from the organisational websites, before initial contacts via email were made to invite them to participate in the research. Upon receiving their agreement, each participant was invited to a semi-structured interview via Zoom, which lasted from 30 to 60 minutes. Pseudonyms are used and participants’ organisations are kept anonymous to protect their identity.

**Table 1.** Participants’ demographics.

No.	Name of participants (pseudonym)	Gender	Position/Role	Type of organisation
1	Wendy	F	Education consultant	Private consultancy org.
2	Tom	M	Project manager	Government org.
3	Tristian	M	Education analyst	Non-government professional org.
4	Tyler	M	Senior officer	Government org.
5	Victor	M	Education analyst	Non-government professional org.
6	Jack	M	Education analyst	Non-government professional org.
7	Anna	F	Director of education analysis	Private consultancy org.
8	Trent	M	Education consultant	Non-government professional org.
9	Caroline	F	Principal advisor, tertiary education	Private consultancy org.
10	Paul	M	Education advisor	Private consultancy org.
11	Jo	F	Education analyst	Non-government professional org.
12	Daniel	M	Executive leader	University
13	Stephen	M	IE leader	University
14	Frank	M	Academic	University
15	Andy	M	Academic	University
16	James	M	Manager	University
17	Carol	F	Project administrator	Non-government professional org.
18	Amelia	F	Head	Non-government professional org.
19	Bianca	F	Head	Non-government professional org.
20	Demi	F	Project administrator	Non-government professional org.
21	Barbara	F	Manager	University
22	Travis	M	Head	Non-government professional org.

The researchers used NVivo software version 12 as a tool for open ending categorisation of the raw data and adopted the constant comparison approach (Denzin and Lincoln, 2000) to support the thematic analysis of the data.

Informed by the research question and literature review, we first identified the key first-order themes for the data to be coded and for the codebook to be developed, including impacts of geopolitical crisis, university response and recommendations. The codebook that guides the categorisation of the raw data was used as a priori approach, allowing specific codes to emerge from the data under the three broad themes. The codebook kept segments of similar texts to help with thematic analysis while capturing participants’ diverse perspectives under specific themes. We highlighted the phrases, sentences and paragraphs from the interview transcripts that align with three broad themes of impacts, responses and recommendations. We compared each of the phrases, sentences and paragraphs emerging from the interview transcripts in relation to these three areas with all the other codes. These codes were then classified to create categories under each of the three themes (see Table 2 for the final codebook summarising the themes, categories and codes). Quotes that most typically represent participants’ voices under specific categories were selected for the analysis. In short, the key points were identified and developed through a thorough process of engagement with the interview excerpts, interpretation of themes in light of the conceptual framework and relevant literature. This represents a process of constant comparison of data (Denzin and Lincoln, 2000) which served as a tool to assist with the reliability of empirical data analysis and support the study’s credibility.

**Table 2.** Final codebook.

	Theme	Category	Codes
Research question 1	Impact of geopolitical crisis on IE and universities	Impact on international student recruitment and university revenues	• International students’ decision under the impact of unwelcoming message
			• International students’ decision under the impact of border closures
			• Role of family influence on students’ decision-making
		Impact on well-being of international students	• Racism
			• Feeling of insecurity
			• Xenophobia
			• Financial hardship
			• Mental health problems
		Impact on other aspects of IE	• Reputation of Australian IE
			• International collaboration
			• International research partnership
			• Transnational education
			• Outbound mobility
Research question 2	Responses of Australian IE and universities to geopolitical crisis	Federal government level	• Slow response
			• Lack of support for and understanding about International education and HE sector
			• Lack of care and sympathy for international students and university staff
			• Hawkishness to China and Chinese students
		State government level	• Difference between state and federal government and among states
			• Support for international students (housing, food, mental health, legal support)
		University level	• Diversifying student cohort
			• Digitalisation of teaching and learning
			• Adjustment of tuition fees
			• Risk management
			• Maintaining institution-to-institution relationships
			• Lack of funding and resources to support international students
			• Staff’s lack of expertise to support international student under impact
			• Differences across universities
Research question 3	Recommendations for Australian IE and universities to improve responses to geopolitical crisis	Reciprocity	• Value of partner countries to Australian IE
			• Australian contribution to partner countries
		Collaboration and inter-relation	• Collaboration between government levels and departments, stakeholders, and dimensions of IE
			• Switch from competitive to collaborative model
		Responsiveness	• Risk assessment and management
			• Dealing with changing needs, trends, challenges, and opportunities
		Empathy	• Care for and inclusion of international students
			• Capacity building for universities in supporting international students
			• Empathy for university staff

The following discussion analyses key stakeholder perceptions as to the issues and possible responses required to revitalise international education in Australia.

## Discussion of data and findings

Crisis is often construed to be a problem to be resolved and perceptions of crisis are impacted by how it is communicated and acted on. ‘Indecision, inaction or inertia, and generally letting the crisis run its course are seen as economically and politically costly, socially unjust, etc’. (De Rycker and Don, 2013: p. 3). But crises are products of discourses that have material effects in terms of actions taken (or not taken) in the context that’ we live in a world that has to make decisions concerning its future under the conditions of manufactured, self-inflicted insecurity’ (Beck, 1999, p. 8). The themes which emerged from the stakeholder data were clear that the rapidly shifting geopolitics of the region and the pandemic, and the discursive and policy responses by the former Australian government to both, created a conjuncture of interrelated events or of multiple crises which impacted on international education.

### Impacts of geopolitics on Australian international education

Geopolitical tensions together with the pandemic have created turbulences for higher education (Lucas, 2021; Maher and Van Noorden, 2021; Ross, 2020b) that have increasingly necessitated the reimagination of the current operating model. While the impacts of geopolitics on IE are visible, the participants interviewed in this study largely agree that geopolitical tensions alone cannot damage the IE sector as it has always been vulnerable to global crises and domestic issues and revived. However, geopolitics is the key factor that ratchets up the level of impact when coupled with an inadequate response of inaction by the federal government to support universities and students during the pandemic. The federal government, despite being responsible for the university sector, showed significant indifference to the HE funding crisis exposed by COVID-19 and indeed exacerbated the crisis and worsened the discursive positioning of international students (Blackmore 2020; Tran, 2020b). First, the former Prime Minister told international students unable to support themselves financially to go home when international borders were closed in March 2020. Second, the previous federal government then actively excluded universities from JobKeeper which would have provided financial protection for staff and excluded international students who worked from JobSeeker (which provided support for those seeking employment) as non-citizens (Tran, 2020b). Third, the government radically changed funding to the HE sector through the Job-ready Graduates legislation which has doubled fees for the Arts and Business (the latter where international students are concentrated) and halved fees for the sciences, overall reducing per-capita funding for the sector (Australian Government, 2020). These moves followed from their ongoing ideological stance aiming to vocationalise higher education and reduce its critical edge (Blackmore, 2020).

At the same time, the heightened discourse by the government about China also shifted public attitudes towards Asian students as captured below by a private consultant’s comment.

... if you’ve got that geopolitical tension happening, but at the same you’ve got students reporting heightened racism and really feeling unsafe and not having access to clear and consistent information and so forth, that’s when you’ve got a problem. *So, the two things happening together is where the problem lies.* If you’ve got one of those things happening, it’s not quite as severe. .... [B]ut I would say that [geopolitics is] certainly a key part of creating *this perfect storm*. (Anna, Director of Education Analysis, private consultancy org.)

Students and their families’ choice of study destination is informed by their ‘subjective’ perception of risks and individual risk calculation (Beck, 2013: p. 10). In calculating the potential risks involving a certain destination, students and families take into consideration multiple interrelated factors apart from geopolitical risks, including health security, racism, xenophobia, the treatment of international students and visa and border policies as remarked by participants in this study (e.g. Jack and Amelia). Hence, in the midst of the geopolitical crisis and securitisation logic played by both home and host countries (He, 2019; Hurst, 2021), the image of Australia portrayed through political statements, the media, communication from agents and within student communities, is a crucial factor contributing to shaping students’ decisions.

Meanwhile, the lack of support and empathy for international students and the border closure policy of the former federal government together with difficulty obtaining visas together with the Chinese government’s position has significantly discouraged Chinese and other international students from choosing Australia as a study destination.

And so, when you start having, for example, federal politicians, basically calling people – well, you know, being suspicious of anybody that looks Chinese, that doesn’t really help for Australia’s image perspective, ... *it’s almost like telling them that “we don’t really welcome you, so don’t come.”* (Wendy, education consultant, private consultancy org.)

*I think Australia’s done some things that I can’t see the necessity for* - some visa cancellation, and the unforgivably slow processing of some research student visas, including Chinese research student visas. I think it’s unjustifiable. And I think Australia should do better in that score. So, I think there’s fault on both sides. (Jack, education analyst, non-government professional org.)

The Australian government therefore created a public discourse that imparted an unwelcoming message towards international students by asking them to go home, closing the border, and being slow in visa processing. At the same time, incidents of racism, xenophobia, financial hardship and mental health problems experienced by international students in Australia, particularly Asian students, as commented by some participants (e.g. Carol, Travis and Bianca) documented in official reports such as the COVID-19 Racism Comprehensive Report (Asian Australian Alliance, 2021) and in the media (e.g. Yang, 2021) indicated the discursive effects of this stance. These factors, coupled with increased geopolitical tensions between Australia and China, exert a combined detrimental impact on the reputation of Australian international education and therefore have been an obstacle for the recovery of the sector. In June 2022, Australia’s recruitment of Chinese international students was down 16% on 131,443 (Australian government, 2022).

### Government responses to geopolitics-pandemic crisis in international education: ideological, ad hoc and inconsistent

The thematic analysis revealed inconsistent and conflicting approaches to handling the dual geopolitical-pandemic crisis across different levels in Australia, that is, federal government, state and territory government and institutions. Responses of Australian previous federal government are believed to be slow, reactive, and unsympathetic as compared to other key destination countries such as the UK, Canada and New Zealand who continued to recruit international students despite the pandemic. However, this response aligned with the Morrison government’s initial reluctance, to put health before the economy and manage COVID-19 to ensure health safety. This belated response to the COVID-19 crisis was not matched in higher education. The Morrison government’s ongoing lack of support for international students and indeed universities (Blackmore 2020; Tran, 2020) was recognised by most of the stakeholders as part of a confrontational attitude towards universities. As an international education university leader remarked,

[M]y sense is that the Australian government’s been almost deliberately slow to revive the international education industry. But, *in some ways that fits with a larger picture whereby it’s managing COVID-19 by prevention and by isolating the Australian population from the virus*... Leaving aside the question of the Australian government’s attitude to international education and I think its desire to run down the finances of the universities and weaken them. (Stephen, IE leader, university).

Border closure indicated the Australian government prioritised public health before international education. But nearly two years later, due to pressure from the international education sector and Universities Australia, has seen the revival of international student mobility with the first groups of students arriving after 15 December 2021. Prior to that, alternative possibilities suggested and reported in the media and elsewhere of developing protocols of controlled quarantine on campuses were never adopted. This can be attributed to the hostility of the government towards the higher education sector compared to the financial support given to other sectors (e.g. business and construction industry) and merely amplified the structural issue and long-term policy of reduced funding for the sector and not addressing the failed model of university funding reliant on international students. This was echoed by some participants:

*We didn’t get much traction on the federal level. By the way, the federal government is also hostile to the higher education sector.* That’s an ideological position. It’s so interesting that there’s a hostility to the knowledge-based, inquiry-based sector. And that’s because maybe you don’t want to be open to inquiry and critical analysis. (Amelia, head, non-government professional org.)

*The Commonwealth Government in particular hasn’t been very sympathetic to international students and hasn’t done very much at all to help them in a concrete way*. And that’s been quite different from the way other national governments have behaved, I think particularly in New Zealand, and Canada, and possibly the UK... *And I think the universities have worked hard to be sympathetic to international students. And I think state governments have done quite a bit.* (Jack, education analyst, non-government professional org.)

While the federal government is unsupportive to universities and therefore IE, the local, state and territory governments and universities have demonstrated more sympathy for international students who are experiencing difficult times during geopolitical-pandemic crisis, with certain types of support, such as COVID rental relief in Victoria (Victoria Housing, 2021), International Student COVID-19 Crisis Accommodation Scheme in New South Wales (Service NSW, 2020), as well as free food hampers, legal support and mental health support.

#### Different responses to managing risk

The discourse of crisis and national security mobilised by the Morrison government magnified the different positioning of universities and the government in dealing with international education and collaborative relationships with China. This was exemplified with the foreign interference legislation which established federal overview of all international research collaborations, the unnamed target being China, but with significant effects on universities who have multiple arrangements with Chinese universities in teaching and research (Mulder, 2022). Despite this policy, Australian universities are still keen to continue partnering with Chinese institutions and academics.

I think what I’m learning is *there’s a difference between government narrative and the institution [ones]* ...policy and practice, right? And it will be naïve to say, “Oh, China said we’re not going to work with Australia, therefore we just stop”. The institution will say, “Okay. I’ll go and speak to ... China and say, are we still good?” “Yeah, we’re good.” “Okay, carry on.” So, we just carry on. Unless there’s a real clampdown. (James, manager, university)

This participant pointed to the ideological difference between governments in both countries and universities in the way they perceive risks relative to what they consider to be their primary role, for example, doing research. Research is a global enterprise based on transnational collaboration, as COVID-19 showed in terms of identifying the strain and developing vaccines. At the political level, the Morrison government has taken a more confrontational position with China and tightened its relationship with the US by mobilising a confrontational discourse, even suggesting war (White, 2022). Universities, while paying more attention to the concern about security in cross-border research partnerships and international education activities, still value the institution-to-institution and people-to-people connections with China and perceive this as core to sustainable international education and international research.

At the same time, they are aware of heightened risk factors around cyber security, with the hacking into the Australian National University (ANU) website in 2019, in which China was named, as a warning. Universities, in managing risk, have also become more careful regarding the soft power of Confucius Institutes (funded by China) which many universities have adopted and addressing the subtle influence being brought to bear on Chinese international students on campus and Australian academics in terms of any critical analysis of China by stating values of academic freedom (Hunter, 2019). Academics with US–China research affiliations have decreased and there has been a slowing of international research collaborations (Maher and Van Noorden, 2021).

In the absence of support from the federal government, universities have made continuing effort to deal with the geopolitical-pandemic crisis and risk associated with their reliance on China as the major source of international students. Strategies included diversifying student cohorts to make up for the loss of the Chinese market, e.g., India, Vietnam. Indonesia (Mulder, 2022), supporting international students, and shifting to teaching and learning online. While diversification has been in university strategic plans for some time, the challenge has been to find substitute markets equivalent in size to China with China also emerging as a competitor in the Asian market.

No, I don’t think they have [effectively diversified]. *But to effectively diversify to make up for a major loss of Chinese students is extremely difficult, if not impossible the way things stand at the moment because you can’t just create a large market for international education if that’s not already there*. (Tristan, education analyst, non-government professional org.)

Although international education is often seen to be market driven, as argued earlier, international student mobility is often based on developing partnerships and infrastructure in-country to gain the benefits of scale. This requires significant planning, time and effort, often developed from research collaborations and institutional partnerships as well as reputation building in specific regions.

With COVID-19, universities, in taking up the slack of the federal government, are becoming increasingly active regarding pastoral care for their international students, although still largely approaches are *ad-hoc* and inconsistent across the sector. Wendy (consultant) notes that while some universities have reduced tuition fees for international students to compensate for the pivots to online learning during the pandemic and cater for students’ financial struggles, others still charge full fees.

*I must say some universities are* doing *better than others, and they spend more effort in addressing it*... But at the same time, I’ve also heard that students have been chased for their tuition fees, for example, and part of me just thinks, they’re actually studying overseas right now, rather than being on campus, and some students might actually be struggling, *so how much universities are actually supporting them in that process, is quite minimal*. (Wendy, education consultant, private consultancy org.)

Most notably, moving online has been the university response to both geopolitical and pandemic crisis. However, as James indicated, some universities that have had more experience in blended learning are more adaptive than others. Online provision requires significant digital infrastructure and academic support that requires a cultural shift in teaching and learning.

The universities that had online already and invested heavily are pivoted really, really well. The ones that haven’t are struggling and having to spend a lot of money. (James, manager, university)

This has led to significant effects on international student experience which is also being recognised by state and federal governments. But overall, there has been variation and fragmentation across the sector including universities in their effort to return to normal as international students begin to arrive. This signifies the need for a more holistic and consistent response across the sector to regain the prior reputation for Australian international education and trust from the international communities.

### Moving forward: the need to build a reciprocal, coordinated, responsive and empathic international education sector

The perspectives of participants in this study advocate for the critical need for the Australian IE sector to work towards a reciprocal, coordinated, responsive and empathetic approach for a sustainable recovery from the dual geopolitics-pandemic crisis.

#### Reciprocal

Participants emphasised that for the Australian IE sector to navigate through geopolitical turbulences, there is a need to develop more reciprocal understandings and learning between Australia and partner countries in the space of inbound and outbound student mobilities, research collaboration or transnational education (TNE). The reciprocity should place emphasis on adding mutual values for Australia and partner countries alike, as commented by Anna, Paul and Trent below.

I think *if we could be getting more young Australian people interested in China, interested in understanding China and interested in going to China, that serves a few purposes*. Because it’s actually – it’s helping to be humble and to be able to say, we would like to learn from you at the same time…. (Anna, Director of Education Analysis, private consultancy org.)

We have had almost an aid view of the world. ... *And we’ve got to get out that technology or a vision, and really think about where we are today... in terms of ... how we can benefit the provider and... add value to them... We desperately need to be more international or global in our perspective.*.. (Paul, education advisor, private consultancy org.)

*It isn’t just knowing that, it’s* understanding *how the political system impacts on what’s delivered on the ground*… So sometimes the difference in geopolitical systems is what you want because you’re going to learn something from that. (Trent, education consultant, non-government professional org.)

As these excerpts suggest, Australians need to switch from the mindset of aid and Western superiority to a more global perspective in which Australia is part of a wider global community and regional network . It also requires politicians to shift from a more confrontational discourse to one that addresses geopolitics of the region in a more subtle manner, which the Labour government appears to be doing. Especially, countries in the Asia-Pacific region, the latest site of geopolitical tension being the Pacific nations, for example, Solomons (Kabaoutoulaka, 2022) are important partners not only as the home countries of the large international student population to Australia but also host countries for Australian students studying overseas and important partners in research collaboration and TNE (Tran and Bui, 2021) and in addressing global risks such as the pandemic. As also highlighted in Liyanage et al. (2018), reciprocity is core to understanding and developing social relations in that it opens up new opportunities for learning and transformation. In this sense, developing reciprocal relationships is vital for the sustainability of Australian international education.

#### Coordinated

As discussed above, there exists inconsistency and mismatch in the response to the geopolitical-pandemic crisis at different levels and by different stakeholders including universities and government, indicating the lack of coordination and consultation, even antagonism with the previous government, across the sector and between the sector and government levels and departments. Participants concurred that international education needs to follow an interrelated and collaborative approach instead of each level, stakeholder and dimension operating in silos and that they needed to share expertise and practices that worked with regard to international students. The agency of crisis management is subject to an amalgam of institutional and human agencies so the collaboration between institutional agency, linked to the ability of units and authorities to respond promptly and responsibly to a crisis, and human agency, related to the willingness and ability of individual members to act and collaborate, is critical in crisis management (Oleksiyenko et al., 2022).

As recommended in the excerpt below, different stakeholders such as federal government, state/territory/local governments, universities and community organisations should come together to more effectively leverage the resources and create compound effects on reviving the sector.

Bottom line is, that Australia if it wants to be and get back to a position, ...*we absolutely need all the sectors working together as one*. You need, at the moment we’ve got states controlling different things. We’ve got Austrade running in a different direction. We’ve got governments playing in the education space. We’ve got *different universities with different policies and strategies*. We need to be engaged as a sector. (Paul, education advisor, private consultancy org.)

Within the higher education sector, participants also pointed to the need for universities as a sector to switch from a competitive model to a more coordinated one, marketing as a sector not as individual universities competing for students.

*[I]f universities can pull together an amount of money to provide services to international students... so you can actually provide substantial services* when *you pull together funding*, but universities don’t like that, and not willing to, as well..., and sometimes you think “well are universities actually wanting to support students, or they just want to pretend to support international students. And if they do support international students in a much better way, a lot of this wouldn’t necessarily happen. (Barbara, manager, university)

*[University should]* collaborate*. I don’t see why they have to compete.* The commodification of education has led to competition and people don’t have to mindlessly participate... You don’t have to participate in the competition. Some competition is counterintuitive and counterproductive to generating knowledge and outcomes. (Amelia, head, non-government professional org.)

Well, it is difficult for the sector as a whole because it is a very divided, competitive sector, I suppose, *that everyone is competing with each other.* But there are certain signals that can be [sent]… in collaboration with government. (Anna, Director of Education Analysis, private consultancy org.)

The competition among universities is the result of processes of corporatisation and commodification (Blackmore, 2020), encourages universities to compete over international student recruitment and research funding in order to improve international rankings, which in turn are a key factor in international student choices of study destination. The business-like model dominating the university funding does not offer an answer to the ‘new risks’ arising from multiple global crises. Therefore, it is important for international education in Australia to move beyond the transactional model that puts too much focus on ‘the numbers game’ (Frank, academic) of ranking.

On an international level, collaboration and partnership with other countries should, various stakeholders argued, also be maintained, and strengthened based on institution-to-institution and people-to-people relationships.

*As an example, while it may be that Beijing and Canberra are experiencing tension, it doesn’t mean that people of China and Australia are necessarily experiencing tension and that* the value is to keep those connections. The value within a risk assessed environment ... That’s the role the university plays. (Barbara, manager, university)

*So at an institution-to-*institutional *level, we tend to just keep our relationships because we know anything above us could change very quickly*.... (James, manager, university)

Both Barbara and James highlighted the importance of the relationships at the institutional and personal levels as key to sustainable partnership despite turbulences in national political relations. Tensions in the bilateral relationship between Australia and China, for example, cannot impede universities and academics in the two countries from maintaining collaboration and connection, as exemplified by how intellectual leadership continued during and in response to the pandemic. International institutional collaboration and partnership lay a foundation for student and staff mobilities, international student recruitment, research collaboration as well as TNE, and thereby being beneficial to the recovery of the sector in Australia. Therefore, different dimensions of international education should be interrelated and built upon each other rather than operating in silos.

#### Responsive

To increase institutions’ readiness for modern risks, there is a need for them to take ‘anticipatory precautionary measures’ (Beck, 2013: p. 11) that are embedded in governance practices. Amidst the dual geopolitical-pandemic crisis, the study participants recommended Australian universities develop a more proactive approach to risk management.

When we look at institutions, *we do due diligence*. And part of our due diligence will be, we [have] the right match as a partner, from a branding, from a marketing, from a financial, from a student, staff security?’, “Can we send staff and students out to a country?,” for example, Iran, is probably not a country we’re going to send staff and students to and setup an institute at the moment because of the risks, political risk, health risks.... and that’s where we will absolutely use Austrade, DFAT... we might get independent consultants. (James, manager, university)

We have an *international risk advisor*. So we have a company called International SOS who are contracted to us. ... In the event that anything happens to any of our staff or students offshore, International SOS always will respond on our behalf. And that’s a big part of our risk management… (Barbara, manager, university)

Beck (2013) posits that we are living in a world risk society because of unforeseeable consequences of decisions. Even when new knowledge can transform ‘unpredictable’ risks into “calculable” ones, in such transformation process, it ‘gives rise to new unpredictabilities, forcing us to reflect upon risks’ (Beck, 2013: p. 15).

#### Empathetic

As emphasised by participants, what is crucial to a sustainable and ethical IE sector is the support and empathy for as well as inclusion of international students. While the care factor has been outlined in government policy documents such as the newly released Australian Strategy for International Education 2021–2030 (Australian Government, 2021), it is important that this is made visible on the ground level. As recommended in the excerpts below, there should be changes in the ways international students are treated and valued in Australia:

My recommendation is that don’t treat Chinese students as an extension of the Chinese Communist Party. If you want to support democratic participation and changes in Chinese society back home, then please *do a better job of the inclusion, the support, and the diplomacy that’s required in order for Chinese students to have a good experience of education here*, and to understand democratic processes and the importance of participation in civil society. (Amelia, head, non-government professional org.)

I don’t know what the solution is, but I think *being empathetic, really empathetic to students in different cultural backgrounds is going to be essential* when making policy decisions from now on. (Anna, Director of Education Analysis, private consultancy org.)

These stakeholders recognise how international students are caught up in the geopolitics-international education crisis and domestic politics and need to be embraced, welcomed and supported in an inclusive society and community. The well-being of students is central to them having good experiences while studying in Australia and is more likely to provide them with a sense of belonging and security. Indeed, support, care and empathy for international students are necessary for Australia to restore the reputation of international sector, which has taken Australia decades to build but been quickly damaged by federal government’s careless message that asked international students to return to their home countries during the pandemic (Ross, 2020b). Again, shared values between government, institutions, Australian public and media are needed to make international students welcome.

At the university level, there should be more investment in building staff capacity in relation to supporting international students and taking care of their well-being.

*I think that there is a big* problem *with regards to university support system*. But whenever, for example, even if we direct them to other like study clusters or Commonwealth departments or things like that, they always say, “ask your university.” Your university or education providers are supposed to give you the support. I don’t know where else students can go to when their university do not provide those kinds of support, they are supposed to. (Bianca, head, non-government professional org.)

As universities are the direct point of contact for international students, they should take lead in providing support and duty of care to their students. However, as Bianca remarked, universities do not always have sufficient expertise to assist students in crisis, indicating the need for universities to develop the capacity for staff in this space.

Apart from students, academic staff also need support and protection from universities and government. Currently, academic staff in Australia are not sufficiently protected as compared to other countries such as the US, UK and Europe, where university organisations such as Universities UK, NAFSA (Association of International Educators) and EAIE (European Association for International Education) are active in advocating for academics.

*Australia hasn’t responded well* ... If you look at the US they’ve been very strong in protecting academics from nation states where they’ve been subject to violence or refugee status or protecting their right to research and comment... Same, if you read across EAIE, they’ve done the same within the European Union. There’s a real integration of academic knowledge creation and sharing and protection that sometimes supersedes a nation*. But we haven’t managed to do that.* (Trent, education consultant, non-government professional org.)

I think everyone would respond the same way because it’d be done through the university organisations. Like *Universities UK is stronger than [Universities Australia]---It is better. It’s larger and it’s probably smarter*. It is a very effective organisation, in my opinion. (Stephen, IE leader, university)

This echoes Blackmore’s (2020) critiques about the carelessness of Australian universities to its people and values as they have become corporatized, most evident during the pandemic when over 40,000 academic and professional jobs were lost in 2020 alone and still mounting. Many academics continue to experience heightened workload while living in fear of more job losses as universities are being restructured yet again (Lucas, 2021). If universities are to improve their duty of care to students amidst geopolitical turbulences, it is vital that their academic and professional staff are protected and supported as they are in the front line of working with students. In other words, in order for IE to adapt and mitigate the risks of geopolitical tensions, Australian universities’ international education activities should be guided by a caring culture for students and staff alike.

## Conclusion

International education has proven to be highly prone to geopolitical turbulences. This inter-relation has become crystalised during the pandemic which has accelerated the tensions of Australia–China relationship. Australia’s international education has been undergoing heightened challenges over the last two years, including the drastic drop of international students, suspension in outbound mobility and transnational education, hardship of international students and staff, and the shaken reputation of Australian IE, as reported by the participants in this research. These challenges reflect the ‘modern risks’ facing Australian international education sector that are the result of ‘conscious decisions’ (Beck, 2013: p. 25) made by government, institutions and individual students. However, the findings of this study highlight that while geopolitical impacts are important, these tensions alone cannot damage Australian IE sector. Instead, what is detrimental to international education is the concurrence of geopolitical turbulences and lack of support and sympathy for international students. Such a concurrence is considered as a ‘perfect storm’ damaging the reputation of the sector.

The study provides evidence about the fragmented, inconsistent, and conflicting approaches to responding to the geopolitics-pandemic crisis from key stakeholders in Australia. Participants overwhelmingly agree that there are marked differences in the responses from different government levels to international education and international students. The Australian former federal government is considered by many interviewees to be slow, reactive and unsympathetic to higher education generally and international education in particular, compared to other governments of competing destinations such as Canada and the UK, and also compared to the state/territory and local governments within Australia.

The findings of this study underscore the importance to re-think and rebuild a more *reciprocal, coordinated, responsive and empathetic international education sector* to help Australia regain its positioning in international education. Reciprocity requires Australian universities to understand and learn from other countries as well as care more about other countries’ needs and the values Australia can add to these countries that align with partner needs. In terms of coordination, we recommend re-positioning different dimensions of international education within an institution as being interrelated and built upon each other rather than operating in silos. With regard to being responsive, the geopolitical crisis foregrounds the need for improved risk prevention and management by education providers and government to respond to the risks, changing needs and emerging demands associated with inbound and outbound mobilities, as well as the new trends, challenges and opportunities of transnational education and research collaboration. Finally, being empathetic and caring for international students should be at the heart of an ethical international education sector.

Even though this research provides fresh insights into Australian university response to geopolitics-pandemic crisis, it is limited in its scope with 22 stakeholders being interviewed. In addition, in this study, we could not collect institutions’ formalised risk maps from interviewees as they were confidential resources of their institutions. It would also be worthwhile to have access to this information to enhance our understanding of the mechanism Australian universities have in place to respond to risks and crises.
